# Chicken model of steroid-induced bone marrow adipogenesis using proteome analysis: a preliminary study

**DOI:** 10.1186/1477-5956-8-47

**Published:** 2010-09-14

**Authors:** Sing Chung Li, Ching Yu Lin, Tzong Fu Kuo, Yun Ho Lin, Chia Chun Chen, Way Neng Lin, Wing P Chan

**Affiliations:** 1 School of Nutrition and Health Sciences, Taipei Medical University, Taipei 110, Taiwan, Republic of China; 2 School of Medical Technology and Biotechnology, Taipei Medical University, Taipei 110, Taiwan, Republic of China; 3 Graduate Institute of Veterinary Medicine, National Taiwan University, Taipei 110, Taiwan, Republic of China; 4 Department of Pathology, School of Medicine, Taipei Medical University, Taipei 110, Taiwan, Republic of China; 5 Department of Radiology, School of Medicine, Taipei Medical University, Taipei 110, Taiwan, Republic of China; 6 Department of Radiology, Taipei Medical University-Wan Fang Hospital, Taipei 116, Taiwan, Republic of China

## Abstract

**Background:**

Steroid-induced adipogenesis increases fat-cell volume and pressure in bone marrow. This may be a contributing factor in some forms of osteonecrosis. In this observational study, we aimed to determine the protein expression relating to steroid-induced adipogenesis of femoral bone marrow with use of a chicken model. We compared the histologic features of the femoral marrow of eight methylprednisolone (MP)-treated chickens with those of three control chickens and assessed differential proteins with 2-dimensional gel electrophoresis and differential proteins were identified by MALDI-TOF MS.

**Results:**

One MP-induced chicken died of overdose anesthesia. Methylprednisolone-induced proliferation of adipose tissue and new bone formation were found on histologic examination. In our study, 13 proteins in the control and MP-induced groups were differently expressed and nine protein spots showed marked threefold downregulation after 19 weeks of MP treatment. These were serum amyloid P-component precursor, zinc finger protein 28, endothelial zinc finger protein 71, T-box transcription factor 3, cyclin-dependent kinase inhibitor 1, myosin 1D, dimethylaniline monooxygenase, and two uncharacterized proteins.

**Conclusions:**

Proteomic profiling can be a useful dynamic approach for detecting protein expression in MP-induced adipogenesis of the femur in chickens.

## Background

Osteonecrosis of the femoral head is marked by necrosis of bone and marrow, trabecular bone loss, and fat cell proliferation. Steroid-induced adipogenesis increases fat-cell volume and pressure in the marrow, eventually leading to some forms of osteonecrosis of the femoral head [1-4]. However, the underlying pathobiological mechanism has not been elucidated [5,6] Many investigators have tried, but failed, to establish animal models of steroid-induced osteonecrosis of the femoral head [6-8]. In 1997, Cui and colleagues [2] first reported that significant adipogenesis and trabecular bone loss of the femoral head could be induced by injection of high-dose corticosteroids in a chicken model. Decreased bone morphogenetic protein 2 (BMP2) gene expression was also noted.

One way to understand a disease's pathogenesis and biological mechanisms is by identifying and characterizing individual proteins of interest [9,10]. The proteomic technology of two-dimensional gel electrophoresis (2-DE) has been widely used in chickens [11], pigs [12], rats [13], rabbits [14], and humans [15,16]. This is currently the only technique that can be applied routinely to quantitative parallel expression profiling of large sets of complex protein mixtures [17].

Most previous animal studies have included histopathologic examinations 6 to 20 weeks after corticosteroid treatment [8,18,19]. In rabbits, methylprednisolone (MP) has been shown to increase the incidence of osteonecrosis to a greater extent than prednisolone or triamcinolone [20].

In this observational study, we aimed to use a proteomic approach to determine the protein expression relating to steroid-induced adipogenesis of femoral bone marrow with use of a chicken model, which has not been reported before.

## Materials and methods

A total of 11 white Leghorn female chickens (age, 25 weeks; weight, 2.8 to 3.5 kg) were used. The Institutional Animal Care and Use Committee at National Taiwan University approved the breeding of the animals and the protocol. All animal husbandry and handling followed the standard operating procedures for laboratory animal breeding and management. All chickens were housed in well-ventilated cages, and each was fed with a standard diet (100 g/day).

Chickens were divided into two groups as follows: group A comprised control animals without steroid injection (n = 3); in group B (n = 8), each chicken had MP induction (9 mg/kg; Solumedrol, UpJohn Laboratories, Kalamazoo, MI) via intramuscular injection every other day. Of the eight MP-injected chickens, four had bone marrow aspiration at 12 weeks and at 19 weeks (B1), and the other four had bone marrow aspiration at 19 weeks only (B2). All three control animals had bone marrow aspiration at both 12 weeks and 19 weeks.

### Isolation of bone marrow

Before bone marrow aspiration, the chickens were anesthetized by injection of xylazine (5 mg/kg) and ketamine (25 mg/kg) in the pectoralis major muscle. Approximately 2 cc of aspirate was collected by direct puncture of the proximal femur with a 14 G needle in order to avoid hemolysis. After that the supernatant was collected by centrifugation at 15,000 × g for 15 min at 4°C, and the soluble protein aliquot was stored at -80°C until further analysis.

### Histology

After the aspiration at 19 weeks, all animals were sacrificed by CO_2 _for histological preparation and examination. The femur was dissected and fixed in 10% buffered formalin overnight, decalcified over about 12 hours in 5% formic acid, and then embedded in paraffin. Sections from each specimen (frontal sections, 3 to 5 micron) were prepared with a microtome and stained with routine hematoxylin and eosin. All chicken cadavers were burned at the end of the process. A senior pathologist (Y-H.L) and a veterinary surgeon (T-F.K), both experienced in skeletal histology reviewed all specimens without knowledge of the animals' groups, and a consensus of interpretation was reached. Histologic findings were interpreted as absence (few or subtle) or presence (many or diffuse) of the following features of osteonecrosis in the proximal femur: fat cell proliferation [2,3], trabecular bone loss [3], new bone formation [21], and necrosis of bone and marrow [3,21].

### Two-dimensional gel electrophoresis

This procedure was performed as previously reported [22], with minor modification. The bone marrow samples from group A (n = 3) were combined to form reference gels. The other samples from chickens in group B were also combined for proteome analysis. Both groups were assessed for protein concentration by BIO-RAD Protein assay Kit (BIO-RAD, CA, USA). A total of 200 μg of bone marrow protein, after albumin and IgG removal kit (GE Healthcare, MD, USA), was rehydrated during 16 hours in solution (7 M urea, 2 M thiourea, 4% CHAPS, 2% IPG buffer, 1% DTT). It was loaded with a trace of bromophenol blue onto an immobilized pH gradient (IPG) strip (pI 4-7, 18 cm, GE Healthcare, MD, USA). The proteins were then focused at 300, 1500, 4000, and 8000 V with a total of 54 750 voltage-hours by using an IPGphor IEF System (GE Healthcare, MD, USA) at 20°C. After 20 hours of one-dimensional isoelectrofocusing, the gel strips were equilibrated for 20 minutes in equilibration buffers I (50 mM Tris-HCl, pH 8.8, 6 M urea, 30% glycerol, 2% SDS, and 0.1% dithiotheritol) and II (50 mM Tris-HCl, pH 8.8, 6 M urea, 30% glycerol, 2% SDS, and 0.25% iodoacetic acid). Both incubations were carried out at room temperature with gentle shaking. The second dimension was run according to the PROTEAN II XL vertical electrophoresis cell operating manual (BIO-RAD, CA, USA). A 10% SDS-polyacrylamide slab gel (18 cm) was used for the second-dimension gel electrophoresis. The IPG strip was placed on the surface of the second-dimension gel, and then the IPG strip was sealed with 0.5% agarose in SDS electrophoresis buffer (25 mM Tris base, 192 mM glycine, 0.1% SDS). The gels were separated in 24 mA at 15°C until the bromophenol blue reached the bottom of the gel.

### Gel staining and image analysis

The 2-dimensional analytical gels were stained with a PlusOne Silver Staining Kit, Protein, (GE Healthcare, MD, USA) with a modified protocol to detect proteins. Briefly, the gel was fixed in fixation solution (ethanol/water/acetic acid, 4/5/1, v/v/v) after electrophoresis and treated with sensitizing solutions (0.5 M sodium acetate, 0.5% sodium thiosulphate) for 30 min. After sensitization, the gels were washed and incubated in 0.25% silver nitrate solution for 20 min and then developed by incubation in the developing solution (2.5% sodium carbonate and 0.015% formaldehyde). More than 200 spots could be detected per gel. For image analysis, all silver-stained gels were scanned using ImageScanner II (GE Healthcare, MD, USA). Thirteen consistently differential protein spots from three independent experiments with consistent results involving group A and group B at 19 weeks were excised and subjected to in-gel tryptic digestion. Electronic gel images were exported in tagged image file format (TIFF) and analyzed with PDQuest 2-DE Analysis Software (BIO-RAD, CA, USA). The differential protein spots were loaded onto software for further analysis. Normalized spot volumes were generated from the optical densities in each gel.

### Protein annotation

For identification of the 13 spots, preparative gels with 600 μg protein loaded were stained with SYPRO Ruby Protein Gel Stain (Invitrogen, CA, USA) and matched to the silver-stained analytic gel. The differentially expressed protein spots were excised from the gel and subjected to in-gel trypsin digestion. All protein spots cut from gels were destained with a solution of 15 mM potassium ferricyanide and 50 mM sodium thiosulfate (1:1), washed twice with deionized water and dehydrated in acetonitrile (ACN). The samples were then rehydrated in digestion buffer (20 mM ammonium bicarbonate and 12.5 ng/ml trypsin) at 4°C. After 30 min incubation, the gels were digested by trypsin for at least 12 h at 37°C. The peptide solution was extracted twice using 0.1% trifluoroacetic acid in 50% ACN and dried with N_2_. MALDI-TOF MS analysis was carried out on an ABI 4700 Proteomics Analyzer with delayed ion extraction (Applied Biosystems, CA, USA) at the Proteomics Core Facility (Institute of Biological Chemistry, Academia Sinica, Taiwan) and identified by database searching with peptide masses by using the Mascot search engine http://www.matrixscience.com followed by NCBI database alignment http://www.expasy.ch/mascot.

### Statistical analysis

We used Fisher's exact test to compare histologic features of control and MP-treated animals. A *P *value of less than 0.05 was considered to indicate a statistically significant difference. A Mascot score with *P *< 0.05 was considered statistically significant [9,22]. All excised spots were tested in triplicate for protein identification.

## Results

**Figure 1:**
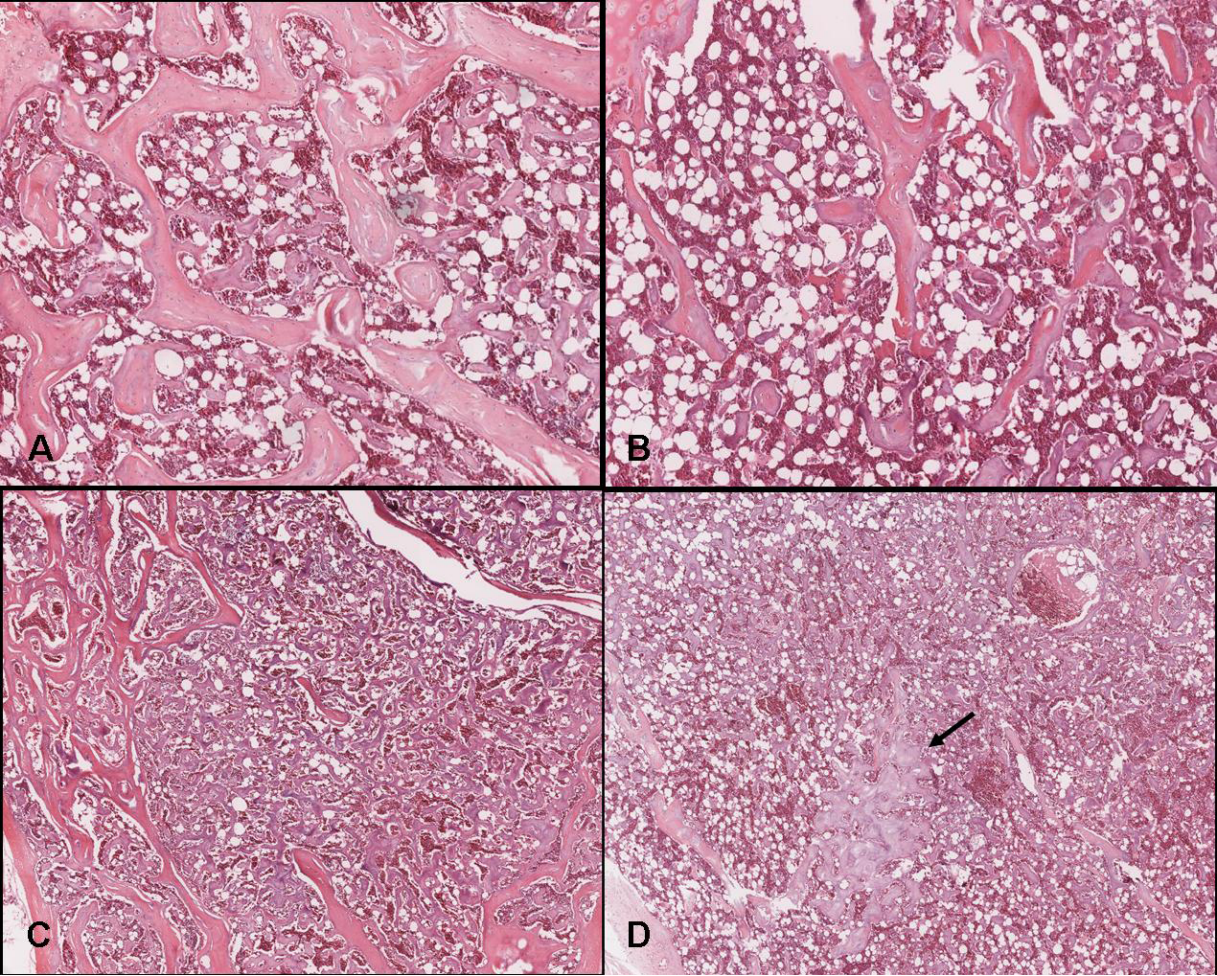
**Photomicrograph of femoral head (A, B) and femoral condylar (C, D) bone marrow**. **(A) **Control chicken at 19 weeks. There are appropriate fatty cells and vacuoles. Thickened primary bone trabeculae with secondary bone trabeculae are seen (H & E). **(B) **Experimental chicken 19 weeks after steroid injection. There is evidence of proliferation of fatty vacuoles. Thinned primary bone trabeculae, with replacement by secondary bone trabeculae are seen (H & E). **(C) **Control chicken at 19 weeks (the same control chicken as in Figure 1A). The section shows fatty cells and bone trabeculae (H & E). **(D) **Experimental chicken 19 weeks after steroid injection (the same experimental chicken as in Figure 1B). The section shows obvious increase in fatty vacuoles. There is evidence of loss of primary bone trabeculae, which have been replaced by marked secondary bone trabeculae with nodular formation (arrow) (H & E).

One chicken in group B2 died of overdose anesthesia during bone marrow aspiration at 19 weeks. Femoral heads and condyles from the remaining seven MP-injected chickens and three control chickens were analyzed histologically. None of the three controls (0/3, Figure 1A), but six chickens in group B (6/7, Figure 1B) showed fat cell proliferation (*P *= 0.03). New bone formation was noted in one chicken from group A (1/3, Figure 1C) and in six from group B (6/7, Figure 1D) (*P *= 0.008). No necrosis of bone or marrow was noted in either group. There were no statistically significant differences between group A (0/3) and group B (1/7) in trabecular bone loss (*P *> 0.05).

**Figure 2:**
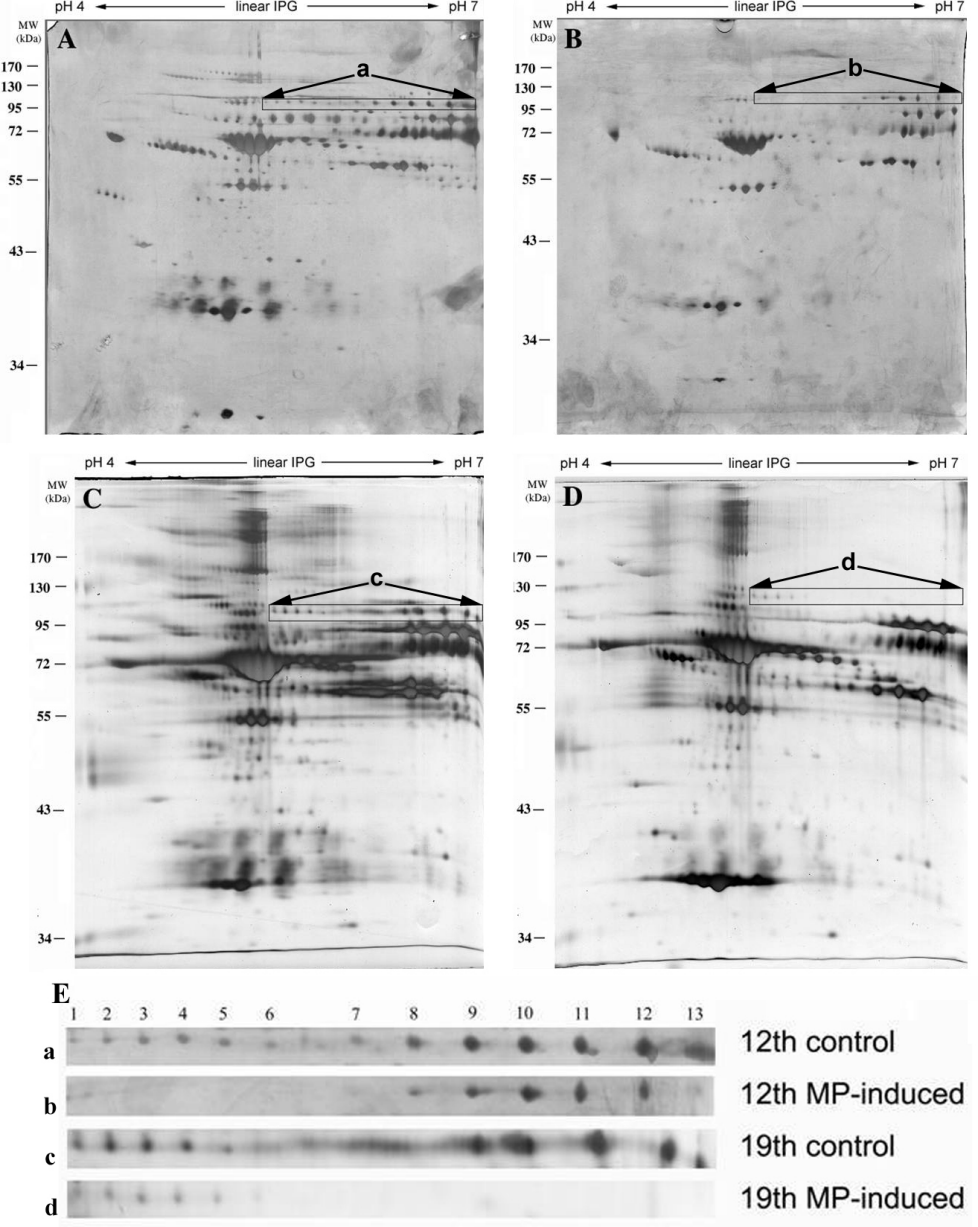
**Representative proteome maps after separation by 2-dimensional gels and silver staining**. **(A) **Control chicken bone marrow at 12 weeks (pooled sample of 3 chickens). **(B) **Steroid-induced chicken bone marrow at 12 weeks (pooled sample of 4 chickens). **(C) **Control chicken bone marrow at 19 weeks (pooled sample of the same 3 control chickens as in Figure 2A). **(D) **Steroid-induced chicken bone marrow at 19 weeks (pooled sample of the same 4 steroid-induced chickens as in Figure 2B). **(E) **Magnification of the proteome maps of (A), (B), (C) and (D). The 13 protein spots shown in the rectangle were further identified by MALDI-TOF MS.

Each 2-dimensional reference gel of control bone marrow obtained at 12 weeks (Figure 2A) or 19 weeks (Figure 2C) was compared with marrow from MP-induced chickens at the same time point (Figure 2B and 2D) for proteome analysis. These proteins were well separated in the 18 cm gel, with an isoelectric point ranging from pH 4 to pH 7. When the 19-week MP-induced bone marrow specimens were compared with control specimens, 13 protein spots showed significant downregulation (Figure 2D and 2E), calculated as the volume ratio of group A over group B (Table 1). These spots had molecular weight around 80 kD and pI range 5-7. Their lowering factors ranged from 0.8 to 2.6 at the 12th week, and from 1.4 to 7.6 at the 19th week (Table 1). The higher adipogenesis during long time MP induction as shown on histology that was in parallel with the 2-dimensional proteome maps at two time interval. We had identified nine proteins (spot 5 to spot 13, Table 1) that were lowered to about a third or less than control volumes after 19 weeks of MP treatment. These matched proteins included serum amyloid P-component precursor, zinc finger protein 28, endothelial zinc finger protein 71, T-box transcription factor 3 (Tbx3), cyclin-dependent kinase inhibitor 1, myosin, dimethylaniline monooxygenase, and two uncharacterized proteins.

**Table 1:** Differentially expressed proteins in femoral marrow of chickens within 12 weeks and 19 weeks after methylprednisolone (MP) induction (pooled sample of 4 chickens), compared with control subjects (pooled sample of 3 chickens).

Spot no.	Protein name	% coverage (score)	Swiss Prot. Accession no.	Matched species	Function	LF in 12th weeks	LF in 19th weeks
1	Coiled-coil domain-containing protein 43	41% (66)	Q5ZK95	Chicken	Evidence at transcript level.	**0.9**	**1.4**
2	Cell death activator CIDE-B	33% (69)	O70303	Mouse	Activates apoptosis [31]	**1.1**	**2.1**
3	Uncharacterized protein C3orf59	17% (61)	Q8IYB1	Human	Unknown	**1.1**	**2.0**
4	Haptoglobin precursor	31% (64)	P00738	Human	Combines with free plasma hemoglobin, preventing loss of iron	**1.4**	**1.7**
5	Serum amyloid P-component precursor	26% (79)	P02743	Human	May be involved in transcriptional regulation [12].	**1.3**	**3.1**
6	Zinc finger protein 28	23% (61)	P17035	Human	May be involved in transcriptional regulation.	**1.2**	**4.1**
7	Endothelial zinc finger protein 71	28% (57)	Q9NQZ8	Human	May be involved in transcriptional regulation.	**1.2**	**5.6**
8	T-box transcription factor 3 TBX3	12% (59)	Q7TST9	Chicken	Transcriptional repressor involved in developmental processes. Probably plays a role in limb pattern formation [9].	**1.0**	**6.2**
9	Cyclin-dependent kinase inhibitor 1	28% (62)	P39689	Mouse	May be the important intermediate by which p53 mediates its role as an inhibitor of cellular proliferation in response to DNA damage. Binds to and inhibits cyclin-dependent kinase activity, preventing phosphorylation of critical cyclin-dependent kinase substrates and blocking cell cycle progression [32].	**0.9**	**7.4**
0	Uncharacterized protein UNQ1940PRO4423 precursor	27% (60)	Q6UWF9	Human	Evidence at transcript level.	**0.8**	**7.5**
11	Uncharacterized protein C12orf52 homolog	39% (66)	Q2HJ75	Bovine	Evidence at transcript level.	**0.9**	**6.6**
12	Myosin	23% (85)	Q17R14	Bovine	Evidence at transcript level.	**1.5**	**7.4**
13	Dimethylaniline monooxygenase	19% (75)	Q8K4B7	Rat	This protein is involved in the oxidative metabolism of a variety of xenobiotics such as drugs and pesticides [29,34]	**2.6**	**7.6**

The lowing factors (LF) were established as the densitometric volume ratio of control over MP-induced group.

## Discussion

In our study, 13 proteins in the control and MP-induced groups were differently expressed and nine proteins were markedly downregulated at 19 weeks. The proliferation of adipose tissue at the 19th week is revealed as a pathologic result of MP treatment. Our mortality rate was lower than that in Cui's study (48%) [2]. The proteomic profiles of the identified proteins were consistently lower at the 19th week in MP-treated chickens, although there was no difference in phenotype. The disappearing proteins may indicate an association with adipogenesis in chicken bone marrow during high-dose MP intervention.

A previous report showed, with use of chicken model, that steroid-induced adipogenesis in the bone marrow may contribute to osteonecrosis of the femur [2]. Adipocytes and osteoblasts share a common pool of fibroblast-like stem cells. When exogenous stimuli, such as steroids, shift the differentiation of marrow stem cells into the adipocyte lineage, the stem cell pool may not be sufficient to provide enough osteoblasts to meet the need for bone remodeling or repair of necrotic bone [1,23,24]. Steroids induce significant fat accumulation in marrow, which contributes to intraosseous hypertension and decreased blood flow [3]. A previous study found large areas of subchondral bone death with new bone formation in 33% of chickens (4/12) 12 and 24 weeks after steroid injection [2]. Similarly, our chickens had significant adipogenesis and new bone formation 19 weeks after MP induction.

At 12 weeks, expression of the proteins did not significantly differ between groups A and B1, except for a ratio of 2.6 in dimethylaniline monooxygenase expression, which increased to 7.6 after 19 weeks (Table 1). Dimethylaniline monooxygenase is one of the flavin-containing monooxygenases, which are related to drug and pesticide metabolism [25], and is involved in xenobiotics metabolism and adaptability in drug response [26].

Most of the 13 proteins have been well described. Govoni *et al*. [27] proposed that Tbx3 is an important determinant of osteoblast cell number. Inoue *et al*. [28] indicated that cyclin-dependent kinase inhibitor 1 is involved in adipocyte differentiation and in protecting hypertrophied adipocytes against apoptosis. The cell death activator CIDE-B is a member of the CIDE family of apoptosis-inducing factors, and overexpression of *CIDE-B *results in cell death associated with the fragmentation of DNA [29]. The amyloid P component (SAP) is an acute-phase serum protein in mice and humans that may serve as a mediator of nonspecific host-defense response [30]. The other proteins, such as coiled-coil domain-containing protein 43, zinc finger protein 28, endothelial zinc finger protein, uncharacterized protein, and myosin 1D may have nothing to do with adipogenesis.

Decreased amounts of haptoglobin precursor, Tbx3, and cyclin-dependent kinase inhibitor 1 protein in MP-treated chickens may be related to adipogenesis. Lerner *et al*. [31] noted that haptoglobin can stimulate bone resorption in osteoblasts, and that the rate of bone resorption in inflammation-induced bone loss may be due to more than a single factor. Lee *et al*. [32] showed that Tbx3 is involved in proliferation and osteogenic differentiation of human adipose stromal cells. Kang *et al*. [33,34] demonstrated that cyclin-dependent kinase inhibitor 1 is involved in adipogenic differentiation of bone marrow-derived human mesenchymal stem cells (hMSCs), and that cyclin-dependent kinase inhibitor 1 has a role in the differentiation-dependent cascade regulating adipogenic differentiation. These three proteins may be synergistically involved with steroid-induced adipogenesis and osteonecrosis in chicken bone marrow [33,34].

In our study, without isolation of osteoprogenitor cell fractions in bone marrow, we could not exclude the possibility that the differential proteins, as identified by MALDI-TOF MS, participate in tissue injury reactions other than adipogenesis in osteonecrosis. It is possible that the biological significance of the differential spots as the main molecules for adipogenesis in the bone microenvironment may have been overstated. Our interpretation will be more certain if the expression of the differential spots as protein markers can be verified by immunoblotting or RT-PCR in a clinical trial of osteonecrosis of the femoral head.

Our study has the limitations that the experimental groups were small and that we did not show a direct association between protein expression and osteonecrosis, but could mimic phenomena following steroid injection. The influence of high-dose MP induction over 12 and 19 weeks in chickens needs further study with a larger sample size. Another important issue to be considered for further studies is although we followed the procedure of Cui's study [2] using female chickens, males may be affected differently; a recent report has indicated that male rabbits may have larger fat cells in the bone marrow than female rabbits after steroid induction [35].

## Conclusions

We have characterized the proteome of extracellular proteins in chicken bone marrow, and have detected and identified nine proteins that were much lower 19 weeks after MP treatment. These altered proteins may be linked to adipogenesis of bone tissue under conditions of excessive glucocorticoid. The proteomics approach in our study may offer a new technique for early detection of potential steroid-induced osteonecrosis of the femur.

## Abbreviations

2-DE: two-dimensioned electrophoresis; BMP2: bone morphogenetic protein 2; CHAPS: 3-[(3-cholamidopropyl) dimethylamonio]-1-propane sulfonate; HMSCS: human mesenchymal stem cells; IPG: immobilized pH gradient; MALDI-TOF MS: matrix-assisted laser desorption ionization time-of-flight mass spectrometry; MP: methylprednisolone; RT-PCR: reverse transcription-polymerase chain reaction; SDS: sodium dodecyl sulfate; TBX3: T-box transcription factor 3; TIFF: tagged image file format;

## Competing interests

The authors declare that they have no competing interests.

## Authors' contributions

Conception and design (SCL, WPC), acquisition of data (SCL, CCC, TFK, YHL, WNL), analysis and interpretation of data (SCL, CCC, TFK, YHL, WNL); drafting the manuscript (SCL, CYL, WPC) and revising it critically for important intellectual content (WPC); and all have given final approval of the version to be published (SCL, CCC, TFK, YHL, WNL, WPC).
